# Editorial: Advances in nutrition, food processing and monitoring

**DOI:** 10.3389/fnut.2023.1179597

**Published:** 2023-04-11

**Authors:** John-Lewis Zinia Zaukuu, Isaac Amoah, George Bazar, László Abrankó, Zoltan Kovacs

**Affiliations:** ^1^Department of Food Science and Technology, Faculty of Bio-sciences, College of Science, Kwame Nkrumah University of Science and Technology, Kumasi, Ghana; ^2^Department of Biochemistry, Faculty of Bio-sciences, College of Science, Kwame Nkrumah University of Science and Technology, Kumasi, Ghana; ^3^Adexgo Kft, Balatonfüred, Hungary; ^4^Department of Food Chemistry and Analysis, Institute of Food Science and Technology, Hungarian University of Agriculture and Life Sciences, Budapest, Hungary; ^5^Department of Measurements and Process Control, Institute of Food Science and Technology, Hungarian University of Agriculture and Life Sciences, Budapest, Hungary

**Keywords:** modeling, nutritional intervention, functional foods, spectroscopy, NIRS

In recent decades, several efforts aimed at providing an understanding of the developing trends of nutrition in an ever-increasing population have been reported. This is mainly because there has been an increased consumer demand for enriched foods, special diets, and functional foods due to growing health concerns. Consumers have become more interested in foods with added value that can be used for nutritional interventions (1).

Consequently, there have been rapid advances in methodologies and technologies geared toward improving efficiency and outcomes in the field of nutrition and food processing. These advances have resulted in the development of several novel methods and portable point-of-care devices whose operations have largely depended on advances in science, involving a blend of biology, chemistry, and physics. Some of these methods and techniques include advanced drying techniques, chromatographic approaches, spectrometry and spectroscopy, sensor-based devices, and nanotechnologies (2).

This editorial provides details about the Research Topic which brought together a list of original papers published on advances made in nutrition, food processing, new food product development, and the use of spectroscopic-based devices for the monitoring of the attributes of these newly developed food products to combat nutritional deficiencies.

Globally, an estimated two billion people suffer from a chronic deficiency of micronutrients (3). Even deficiencies in a mild to moderate context have been reported to be capable of resulting in impaired physical and cognitive abilities, poor physical growth, and work impairments, which could all be considered as hidden hunger. Diet quality, therefore, is an important determinant of the development of diet-related chronic diseases (4). Consequently, there has been a need for the food industry to reformulate foods to improve their healthiness. Csurka et al. researched the prospect of valorizing powdered blood from livestock as a substitute for egg allergen in the preparation of sponge cakes and investigated the physicochemical properties of the cake. The authors reported that cakes enriched with blood products were not different in preference, compared to the control non-enriched counterparts. However, the addition of powdered blood products to cakes increased their hardness, compared to the control sample. The storability properties of the cakes were negatively affected after 3 days of storage. Food processing impacts the matrix composition, resulting in changes in physicochemical properties and could potentially improve the shelf-life of the food.

Tassy et al. investigated the nutritional quality of packaged foods launched globally between 2016 and 2018 and those launched between 2018 and 2020, as reported in the Mintel Global New Products Database. The authors compared these two time periods in order to determine whether there has been recent improvement in food reformulation to improve their healthiness using indicators including proteins, fibers, sugars, saturated fatty acids, and sodium. The authors pointed out that food reformulation to improve the healthiness of packaged food, especially through reductions in the sodium and sugar content was observed in the period under study.

The effect of the processing method on the nutritional composition of eggs produced by chickens has been investigated (Pirkwieser et al.). The authors investigated the effect of pasteurization and spray drying on the nutritional profile of eggs from hens. No changes in total fat content, amino acid profile, α- and δ-tocopherol, lutein, zeaxanthin, essential trace elements, and cobalamin following the use of the spray-drying method were observed. These highlights spray drying as an effective processing method to maintain the nutritional integrity of eggs.

The type of food processing method employed impacts the structural modification of the matrix of the food. This property can be exploited to improve the health-promoting properties of newly developed food products. The effect of the consumption of steamed potato flour used in composite formulation with wheat for bread development on metabolic indices has been investigated. Xu et al. conducted a randomized controlled trial that assessed the effect of the acute intake (4 weeks) of steamed potato-wheat bread on weight, lipids, glucose, and Na+/K+ concentrations. The authors observed significant reductions in body weight, lipid weight (*p* = 0.016), body mass index (*p* = 0.020), low-density lipoprotein cholesterol (*p* = 0.035), and the urinary level of Na+/K+ (*p* = 0.007).

In the food processing and product development space, the use of these devices has resulted in maintaining quality standards in the nutritional, physicochemical, and sensorial integrity of newly developed food products. Near-infrared spectroscopy (NIRS) is one common and essential technique that has been used to track food product quality in recent times. Largely, NIRS devices operate using spectroscopic principles and have a potential for non-invasive and on-field analysis using their handheld/portable versions or in-line setup when automated measurements can be performed. They can therefore serve as a buffer to keep pace with the novel trends of food fortification and product development.

For example, Li et al. established a deep neural network method to classify navel orange surface defects and subsequent automated classifications of fruits with a normal vs. defective surface. They used Standard Normal Variate (SNV) transformation to eliminate baseline drift and an independent component analysis-genetic algorithm to classify the defective surfaces. In their conclusion, wavelength selection was crucial in obtaining good classification accuracies. However, only images from the upper surface of the navel orange were collected, while lower surface images were not. Therefore, a major research focus includes capturing and analyzing images of the entire surface of navel oranges in subsequent online detection work.

Yali pears are fruits with high nutrients and sensory qualities but are often subjected to attacks by pests due to the improper nursing of pear trees. An online rapid non-destructive detection method for internal defects of Yali pears based on Vis-NIR was proposed based on a deep learning model for monitoring Yali pear quality in a bid to guarantee a high level of sorting of the fruits (Hao et al.). The results showed that the online discriminant model established based on spectra pretreated by Savitzky-Golay Smoothing combined with Convolutional Block Attention Module-Convolutional Neural Networks (CBAMCNN) deep learning method yielded the highest accuracy where the calibration set and validation set had values of 96.88 and 92.71%, respectively. The rapidness of the technique was also proven with a prediction time of 0.032 s for a single Yali pear. The deep learning method makes full use of its autonomous feature extraction and learning ability.

In advancing the scope of NIRS for the complex analysis of moist substances such as many foods and food raw materials, emerging data analysis techniques such as aquaphotomics have also been explored. Aquaphotomics is a technique that simply uses the electromagnetic spectrum of water as a molecular mirror for advanced analysis with NIRS. This method was recently applied to monitor the quality changes of the strawberry fruit during storage in a refrigerator with an electric field generator (supercooling fridge, SCF) and without it (control fridge, CF) (Muncan et al.). From their results, strawberries in CF and SCF showed that exposure to an electric field leads to a delay in ripening by around 3 days. This was evidenced by the increased amount of structural, strongly bound water and vapor-like trapped water in the strawberries stored in SCF.

Different regression techniques were compared for the prediction of the total acidity (TA) of intact mangos (5). It was observed that partial least squares regression, support vector machine, and artificial neural networking could all predict TA with good accuracy. However, the highest coefficient of the determination for TA prediction: 0.985 in calibration and 0.943 in prediction was achieved using the artificial neural networks (ANN) approach. ANN also proved to be the best in terms of the RPD index [Ratio of the standard error of Performance (or prediction) to standard Deviation] reaching 4.02.

In conclusion, designing and implementing nutrition-focused interventions have often involved the use of carefully designed methodologies such as randomized controlled trials, case-control studies, and cross-sectional studies. However, achieving good nutrition depends on and encompasses the entire food supply; this is the backbone of food processing and monitoring techniques. Nutrition, food processing, and monitoring have evolved significantly during the last decades in theory and practice, particularly due to urbanization and an increasing global population. NIRS has proven to be a powerful tool as acknowledged by numerous researchers for being non-invasive and non-destructive but still providing rapid feedback. The technique, when applied in tandem with the novel data analysis approaches, promises to be a good method for food quality evaluations. The editors would like to thank all authors and reviewers for their contributions to this Research Topic.

## Author contributions

All authors listed have made a substantial, direct, and intellectual contribution to the work and approved it for publication.
